# Supplementary material for: Resolving the full spectrum of human genome variation using Linked-Reads
Source: Genome Res. 2019 Apr;29(4):635–45. doi: 10.1101/gr.234443.118 (PMC6442396; doi:10.1101/gr.234443.118)
Supplement: Supplemental Material [file supp_gr.234443.118_Supplemental_longranger-2.2.zip › longranger-2.2.tar.bz2!/lr2.2-public/lib/stan/cmdstan/stan/doc/api/html/hierarchy.html]

Stan: Class Hierarchy


|  |  |
| --- | --- |
|  | Stan  2.5.0  probability, sampling & optimization |


- Main Page
- Related Pages
- Namespaces
- Classes
- Files

- Class List
- Class Index
- Class Hierarchy
- Class Members


All Classes Namespaces Files Functions Variables Typedefs Enumerations Enumerator Friends Macros Pages

Class Hierarchy

This inheritance list is sorted roughly, but not completely, alphabetically:

[detail level 12345]

|  |  |
| --- | --- |
| stan::math::accumulator< T > | Class to accumulate values and eventually return their sum |
| stan::gm::add\_conditional\_body |  |
| stan::gm::add\_conditional\_condition |  |
| stan::gm::add\_expression\_dimss |  |
| stan::gm::add\_fun\_var |  |
| stan::gm::add\_function\_signature |  |
| stan::gm::add\_loop\_identifier |  |
| add\_var |  |
| stan::gm::add\_while\_body |  |
| stan::gm::add\_while\_condition |  |
| stan::gm::addition\_expr3 |  |
| stan::gm::arg\_decl |  |
| stan::gm::argument |  |
| stan::gm::categorical\_argument |  |
| stan::gm::arg\_adapt |  |
| stan::gm::arg\_bfgs |  |
| stan::gm::arg\_lbfgs |  |
| stan::gm::arg\_data |  |
| stan::gm::arg\_diagnose |  |
| stan::gm::arg\_hmc |  |
| stan::gm::arg\_newton |  |
| stan::gm::arg\_nuts |  |
| stan::gm::arg\_optimize |  |
| stan::gm::arg\_output |  |
| stan::gm::arg\_random |  |
| stan::gm::arg\_rwm |  |
| stan::gm::arg\_sample |  |
| stan::gm::arg\_static |  |
| stan::gm::arg\_test\_gradient |  |
| stan::gm::unvalued\_argument |  |
| stan::gm::arg\_dense\_e |  |
| stan::gm::arg\_diag\_e |  |
| stan::gm::arg\_fail |  |
| stan::gm::arg\_fixed\_param |  |
| stan::gm::arg\_unit\_e |  |
| stan::gm::valued\_argument |  |
| stan::gm::list\_argument |  |
| stan::gm::arg\_engine |  |
| stan::gm::arg\_method |  |
| stan::gm::arg\_metric |  |
| stan::gm::arg\_optimize\_algo |  |
| stan::gm::arg\_sample\_algo |  |
| stan::gm::arg\_test |  |
| stan::gm::singleton\_argument< T > |  |
| stan::gm::arg\_adapt\_delta |  |
| stan::gm::arg\_adapt\_engaged |  |
| stan::gm::arg\_adapt\_gamma |  |
| stan::gm::arg\_adapt\_init\_buffer |  |
| stan::gm::arg\_adapt\_kappa |  |
| stan::gm::arg\_adapt\_t0 |  |
| stan::gm::arg\_adapt\_term\_buffer |  |
| stan::gm::arg\_adapt\_window |  |
| stan::gm::arg\_data\_file |  |
| stan::gm::arg\_diagnostic\_file |  |
| stan::gm::arg\_history\_size |  |
| stan::gm::arg\_id |  |
| stan::gm::arg\_init |  |
| stan::gm::arg\_init\_alpha |  |
| stan::gm::arg\_int\_time |  |
| stan::gm::arg\_iter |  |
| stan::gm::arg\_max\_depth |  |
| stan::gm::arg\_num\_samples |  |
| stan::gm::arg\_num\_warmup |  |
| stan::gm::arg\_output\_file |  |
| stan::gm::arg\_refresh |  |
| stan::gm::arg\_save\_iterations |  |
| stan::gm::arg\_save\_warmup |  |
| stan::gm::arg\_seed |  |
| stan::gm::arg\_stepsize |  |
| stan::gm::arg\_stepsize\_jitter |  |
| stan::gm::arg\_test\_grad\_eps |  |
| stan::gm::arg\_test\_grad\_err |  |
| stan::gm::arg\_thin |  |
| stan::gm::arg\_tolerance |  |
| stan::gm::argument\_parser |  |
| stan::gm::argument\_probe |  |
| stan::math::array\_builder< T > | Structure for building up arrays in an expression (rather than in statements) using an argumentchaining add() method and a getter method array() to return the result |
| stan::gm::array\_literal |  |
| stan::gm::assignment |  |
| stan::mcmc::base\_adaptation |  |
| stan::mcmc::stepsize\_adaptation |  |
| stan::mcmc::windowed\_adaptation |  |
| stan::mcmc::covar\_adaptation |  |
| stan::mcmc::var\_adaptation |  |
| stan::mcmc::base\_adapter |  |
| stan::mcmc::stepsize\_adapter |  |
| stan::mcmc::adapt\_unit\_e\_nuts< M, BaseRNG > |  |
| stan::mcmc::adapt\_unit\_e\_static\_hmc< M, BaseRNG > |  |
| stan::mcmc::stepsize\_covar\_adapter |  |
| stan::mcmc::adapt\_dense\_e\_nuts< M, BaseRNG > |  |
| stan::mcmc::adapt\_dense\_e\_static\_hmc< M, BaseRNG > |  |
| stan::mcmc::stepsize\_var\_adapter |  |
| stan::mcmc::adapt\_diag\_e\_nuts< M, BaseRNG > |  |
| stan::mcmc::adapt\_diag\_e\_static\_hmc< M, BaseRNG > |  |
| stan::mcmc::base\_hamiltonian< M, P, BaseRNG > |  |
| stan::mcmc::base\_hamiltonian< M, dense\_e\_point, BaseRNG > |  |
| stan::mcmc::dense\_e\_metric< M, BaseRNG > |  |
| stan::mcmc::base\_hamiltonian< M, diag\_e\_point, BaseRNG > |  |
| stan::mcmc::diag\_e\_metric< M, BaseRNG > |  |
| stan::mcmc::base\_hamiltonian< M, unit\_e\_point, BaseRNG > |  |
| stan::mcmc::unit\_e\_metric< M, BaseRNG > |  |
| stan::mcmc::base\_integrator< H, P > |  |
| stan::mcmc::base\_leapfrog< H, P > |  |
| stan::mcmc::expl\_leapfrog< H, P > |  |
| stan::mcmc::base\_integrator< dense\_e\_metric< M, BaseRNG >, dense\_e\_point > |  |
| stan::mcmc::base\_leapfrog< dense\_e\_metric< M, BaseRNG >, dense\_e\_point > |  |
| stan::mcmc::expl\_leapfrog< dense\_e\_metric< M, BaseRNG >, dense\_e\_point > |  |
| stan::mcmc::base\_integrator< diag\_e\_metric< M, BaseRNG >, diag\_e\_point > |  |
| stan::mcmc::base\_leapfrog< diag\_e\_metric< M, BaseRNG >, diag\_e\_point > |  |
| stan::mcmc::expl\_leapfrog< diag\_e\_metric< M, BaseRNG >, diag\_e\_point > |  |
| stan::mcmc::base\_integrator< unit\_e\_metric< M, BaseRNG >, unit\_e\_point > |  |
| stan::mcmc::base\_leapfrog< unit\_e\_metric< M, BaseRNG >, unit\_e\_point > |  |
| stan::mcmc::expl\_leapfrog< unit\_e\_metric< M, BaseRNG >, unit\_e\_point > |  |
| stan::mcmc::base\_mcmc |  |
| stan::mcmc::base\_hmc< M, dense\_e\_point, dense\_e\_metric, expl\_leapfrog, BaseRNG > |  |
| stan::mcmc::base\_nuts< M, dense\_e\_point, dense\_e\_metric, expl\_leapfrog, BaseRNG > |  |
| stan::mcmc::dense\_e\_nuts< M, BaseRNG > |  |
| stan::mcmc::adapt\_dense\_e\_nuts< M, BaseRNG > |  |
| stan::mcmc::base\_static\_hmc< M, dense\_e\_point, dense\_e\_metric, expl\_leapfrog, BaseRNG > |  |
| stan::mcmc::dense\_e\_static\_hmc< M, BaseRNG > |  |
| stan::mcmc::adapt\_dense\_e\_static\_hmc< M, BaseRNG > |  |
| stan::mcmc::base\_hmc< M, diag\_e\_point, diag\_e\_metric, expl\_leapfrog, BaseRNG > |  |
| stan::mcmc::base\_nuts< M, diag\_e\_point, diag\_e\_metric, expl\_leapfrog, BaseRNG > |  |
| stan::mcmc::diag\_e\_nuts< M, BaseRNG > |  |
| stan::mcmc::adapt\_diag\_e\_nuts< M, BaseRNG > |  |
| stan::mcmc::base\_static\_hmc< M, diag\_e\_point, diag\_e\_metric, expl\_leapfrog, BaseRNG > |  |
| stan::mcmc::diag\_e\_static\_hmc< M, BaseRNG > |  |
| stan::mcmc::adapt\_diag\_e\_static\_hmc< M, BaseRNG > |  |
| stan::mcmc::base\_hmc< M, unit\_e\_point, unit\_e\_metric, expl\_leapfrog, BaseRNG > |  |
| stan::mcmc::base\_nuts< M, unit\_e\_point, unit\_e\_metric, expl\_leapfrog, BaseRNG > |  |
| stan::mcmc::unit\_e\_nuts< M, BaseRNG > |  |
| stan::mcmc::adapt\_unit\_e\_nuts< M, BaseRNG > |  |
| stan::mcmc::base\_static\_hmc< M, unit\_e\_point, unit\_e\_metric, expl\_leapfrog, BaseRNG > |  |
| stan::mcmc::unit\_e\_static\_hmc< M, BaseRNG > |  |
| stan::mcmc::adapt\_unit\_e\_static\_hmc< M, BaseRNG > |  |
| stan::mcmc::base\_hmc< M, P, H, I, BaseRNG > |  |
| stan::mcmc::base\_nuts< M, P, H, I, BaseRNG > |  |
| stan::mcmc::base\_static\_hmc< M, P, H, I, BaseRNG > |  |
| stan::mcmc::fixed\_param\_sampler |  |
| stan::gm::base\_var\_decl |  |
| stan::gm::cholesky\_corr\_var\_decl |  |
| stan::gm::cholesky\_factor\_var\_decl |  |
| stan::gm::corr\_matrix\_var\_decl |  |
| stan::gm::cov\_matrix\_var\_decl |  |
| stan::gm::double\_var\_decl |  |
| stan::gm::int\_var\_decl |  |
| stan::gm::matrix\_var\_decl |  |
| stan::gm::ordered\_var\_decl |  |
| stan::gm::positive\_ordered\_var\_decl |  |
| stan::gm::row\_vector\_var\_decl |  |
| stan::gm::simplex\_var\_decl |  |
| stan::gm::unit\_vector\_var\_decl |  |
| stan::gm::vector\_var\_decl |  |
| stan::optimization::BFGSMinimizer< FunctorType, QNUpdateType, Scalar, DimAtCompile > |  |
| stan::optimization::BFGSMinimizer< ModelAdaptor< M >, QNUpdateType, Scalar, DimAtCompile > |  |
| stan::optimization::BFGSLineSearch< M, QNUpdateType, Scalar, DimAtCompile > |  |
| stan::optimization::BFGSUpdate\_HInv< Scalar, DimAtCompile > |  |
| stan::gm::binary\_op |  |
| stan::gm::binary\_op\_expr |  |
| stan::math::detail::bounded< T\_y, T\_low, T\_high, T\_result, y\_is\_vec > |  |
| stan::math::detail::bounded< T\_y, T\_low, T\_high, T\_result, true > |  |
| stan::agrad::chainable | Abstract base class for variable implementations that handles memory management and applying the chain rule |
| stan::agrad::vari | The variable implementation base class |
| stan::agrad::op\_ddv\_vari |  |
| stan::agrad::op\_dv\_vari |  |
| stan::agrad::op\_dvd\_vari |  |
| stan::agrad::op\_dvv\_vari |  |
| stan::agrad::op\_matrix\_vari |  |
| stan::agrad::op\_v\_vari |  |
| stan::agrad::precomp\_v\_vari |  |
| stan::agrad::op\_vd\_vari |  |
| stan::agrad::op\_vdd\_vari |  |
| stan::agrad::op\_vdv\_vari |  |
| stan::agrad::op\_vector\_vari |  |
| stan::agrad::op\_vv\_vari |  |
| stan::agrad::op\_vvd\_vari |  |
| stan::agrad::op\_vvv\_vari |  |
| stan::agrad::partials\_vari |  |
| stan::agrad::precomputed\_gradients\_vari | This is a var implementation class that takes precomputed gradient values |
| stan::agrad::stored\_gradient\_vari | A var implementation that stores the daughter variable implementation pointers and the partial derivative with respect to the result explicitly in arrays constructed on the auto-diff memory stack |
| stan::agrad::chainable\_alloc | A chainable\_alloc is an object which is constructed and destructed normally but the memory lifespan is managed along with the arena allocator for the gradient calculation |
| stan::agrad::LDLT\_alloc< R, C > | This object stores the actual (double typed) LDLT factorization of an Eigen::Matrix<var> along with pointers to its vari's which allow the \*\_ldlt functions to save memory |
| stan::mcmc::chains< RNG > | An `mcmc::chains` object stores parameter names and dimensionalities along with samples from multiple chains |
| stan::io::cmd\_line | Parses and stores command-line arguments |
| stan::math::common\_type< T1, T2 > |  |
| stan::math::common\_type< Eigen::Matrix< T1, R, C >, Eigen::Matrix< T2, R, C > > |  |
| stan::math::common\_type< std::vector< T1 >, std::vector< T2 > > |  |
| stan::gm::conditional\_statement |  |
| stan::contains\_fvar< T1, T2, T3, T4, T5, T6 > | Metaprogram to calculate the base scalar return type resulting from promoting all the scalar types of the template parameters |
| stan::optimization::ConvergenceOptions< Scalar > |  |
| copy\_square\_cholesky\_dimension\_if\_necessary |  |
| stan::math::coupled\_ode\_observer | Observer for the coupled states |
| stan::math::coupled\_ode\_system< F, T1, T2 > | Base template class for a coupled ordinary differential equation system, which adds sensitivities to the base system |
| stan::math::coupled\_ode\_system< F, double, double > | The coupled ode system for known initial values and known parameters |
| stan::math::coupled\_ode\_system< F, double, stan::agrad::var > | The coupled ODE system for known initial values and unknown parameters |
| stan::math::coupled\_ode\_system< F, stan::agrad::var, double > | The coupled ODE system for unknown initial values and known parameters |
| stan::math::coupled\_ode\_system< F, stan::agrad::var, stan::agrad::var > | The coupled ode system for unknown intial values and unknown parameters |
| stan::common::recorder::csv | Writes out a vector as string |
| stan::io::csv\_writer | Writes Stan variables in comma-separated-value format to an output stream |
| stan::gm::distribution |  |
| stan::gm::division\_expr |  |
| stan::gm::double\_literal |  |
| stan::DoubleVectorView< used, is\_vec > | DoubleVectorView allocates double values to be used as intermediate values |
| stan::DoubleVectorView< true, false > |  |
| stan::DoubleVectorView< true, true > |  |
| stan::io::dump\_reader | Reads data from S-plus dump format |
| stan::io::dump\_writer | Writes data into the S-plus dump format |
| stan::gm::elt\_division\_expr |  |
| stan::gm::elt\_multiplication\_expr |  |
| empty\_range |  |
| stan::gm::error\_codes |  |
| stan::error\_index |  |
| stan::gm::exponentiation\_expr |  |
| stan::gm::expr\_type |  |
| stan::gm::expression |  |
| stan::gm::expression\_as\_statement |  |
| stan::common::recorder::filtered\_values< InternalVector > |  |
| stan::gm::for\_statement |  |
| stan::gm::fun |  |
| stan::gm::function\_decl\_def |  |
| stan::gm::function\_decl\_defs |  |
| stan::gm::function\_signatures |  |
| stan::agrad::fvar< T > |  |
| Eigen::internal::general\_matrix\_matrix\_product< Index, stan::agrad::var, LhsStorageOrder, ConjugateLhs, stan::agrad::var, RhsStorageOrder, ConjugateRhs, ColMajor > |  |
| Eigen::internal::general\_matrix\_vector\_product< Index, stan::agrad::var, ColMajor, ConjugateLhs, stan::agrad::var, ConjugateRhs > | Override matrix-vector and matrix-matrix products to use more efficient implementation |
| Eigen::internal::general\_matrix\_vector\_product< Index, stan::agrad::var, RowMajor, ConjugateLhs, stan::agrad::var, ConjugateRhs > |  |
| **grammar** |  |
| stan::gm::bare\_type\_grammar< Iterator > |  |
| stan::gm::expression07\_grammar< Iterator > |  |
| stan::gm::expression\_grammar< Iterator > |  |
| stan::gm::functions\_grammar< Iterator > |  |
| stan::gm::program\_grammar< Iterator > |  |
| stan::gm::statement\_2\_grammar< Iterator > |  |
| stan::gm::statement\_grammar< Iterator > |  |
| stan::gm::term\_grammar< Iterator > |  |
| stan::gm::var\_decls\_grammar< Iterator > |  |
| stan::gm::whitespace\_grammar< Iterator > |  |
| stan::prob::include\_summand< propto, T1, T2, T3, T4, T5, T6, T7, T8, T9, T10 > | Template metaprogram to calculate whether a summand needs to be included in a proportional (log) probability calculation |
| stan::gm::increment\_log\_prob\_statement |  |
| stan::gm::index\_op |  |
| stan::math::index\_type< T > | Primary template class for the metaprogram to compute the index type of a container |
| stan::math::index\_type< const T > | Template class for metaprogram to compute the type of indexes used in a constant container type |
| stan::math::index\_type< Eigen::Matrix< T, R, C > > | Template metaprogram defining typedef for the type of index for an Eigen matrix, vector, or row vector |
| stan::math::index\_type< std::vector< T > > | Template metaprogram class to compute the type of index for a standard vector |
| stan::gm::int\_literal |  |
| stan::gm::integrate\_ode |  |
| stan::is\_constant< T > | Metaprogramming struct to detect whether a given type is constant in the mathematical sense (not the C++ `const` sense) |
| stan::is\_constant\_struct< T > | Metaprogram to determine if a type has a base scalar type that can be assigned to type double |
| stan::is\_constant\_struct< Eigen::Block< T > > |  |
| stan::is\_constant\_struct< Eigen::Matrix< T, R, C > > |  |
| stan::is\_constant\_struct< std::vector< T > > |  |
| stan::is\_fvar< T > |  |
| stan::is\_fvar< stan::agrad::fvar< T > > |  |
| stan::is\_var< T > |  |
| stan::is\_var< stan::agrad::var > |  |
| stan::is\_var\_or\_arithmetic< T1, T2, T3, T4, T5, T6 > |  |
| stan::is\_vector< T > |  |
| stan::is\_vector< const T > |  |
| stan::is\_vector< Eigen::Block< T > > |  |
| stan::is\_vector< Eigen::Matrix< T, 1, Eigen::Dynamic > > |  |
| stan::is\_vector< Eigen::Matrix< T, Eigen::Dynamic, 1 > > |  |
| stan::is\_vector< std::vector< T > > |  |
| stan::is\_vector\_like< T > |  |
| stan::is\_vector\_like< const T > |  |
| stan::is\_vector\_like< Eigen::Matrix< T, Eigen::Dynamic, Eigen::Dynamic > > |  |
| stan::is\_vector\_like< T \* > |  |
| stan::json::json\_handler | Abstract base class for JSON handlers |
| stan::json::json\_data\_handler | A `json_data_handler` is an implementation of a `json_handler` that restricts the allowed JSON text a set of Stan variable declarations in JSON format |
| stan::optimization::LBFGSUpdate< Scalar, DimAtCompile > | Implement a limited memory version of the BFGS update |
| stan::math::LDLT\_factor< T, R, C > |  |
| stan::math::LDLT\_factor< stan::agrad::var, R, C > | A template specialization of src/stan/math/matrix/LDLT\_factor.hpp for stan::agrad::var which can be used with all the \*\_ldlt functions |
| stan::math::LDLT\_factor< T, R, C > | LDLT\_factor is a thin wrapper on Eigen::LDLT to allow for reusing factorizations and efficient autodiff of things like log determinants and solutions to linear systems |
| stan::gm::left\_division\_expr |  |
| **logic\_error** |  |
| stan::json::json\_error | Exception type for JSON errors |
| stan::gm::logical\_negate\_expr |  |
| stan::optimization::LSOptions< Scalar > |  |
| stan::io::mcmc\_writer< M, SampleRecorder, DiagnosticRecorder, MessageRecorder > | Mcmc\_writer writes out headers and samples |
| stan::common::recorder::messages |  |
| stan::model::model\_functional< M > |  |
| stan::optimization::ModelAdaptor< M > |  |
| stan::gm::modulus\_expr |  |
| stan::gm::multiplication\_expr |  |
| stan::gm::negate\_expr |  |
| stan::gm::nil | Placeholder struct for boost::variant default ctors |
| stan::common::recorder::no\_op | Does nothing |
| stan::gm::no\_op\_statement |  |
| stan::common::NoOpFunctor |  |
| std::numeric\_limits< stan::agrad::fvar< T > > |  |
| std::numeric\_limits< stan::agrad::var > | Specialization of numeric limits for var objects |
| Eigen::NumTraits< stan::agrad::var > | Numerical traits template override for Eigen for automatic gradient variables |
| stan::mcmc::nuts\_util |  |
| stan::agrad::OperandsAndPartials< T1, T2, T3, T4, T5, T6, T\_return\_type > | A variable implementation that stores operands and derivatives with respect to the variable |
| stan::math::pass\_type< T > |  |
| stan::math::pass\_type< double > |  |
| stan::math::pass\_type< int > |  |
| stan::gm::print\_statement |  |
| stan::gm::printable |  |
| stan::model::prob\_grad | The `prob_grad` class represents the basic parameter holders for a model |
| stan::gm::program |  |
| stan::math::promote\_scalar\_struct< T, S > | General struct to hold static function for promoting underlying scalar types |
| stan::math::promote\_scalar\_struct< T, Eigen::Matrix< S, 1,-1 > > | Struct to hold static function for promoting underlying scalar types |
| stan::math::promote\_scalar\_struct< T, Eigen::Matrix< S,-1, 1 > > | Struct to hold static function for promoting underlying scalar types |
| stan::math::promote\_scalar\_struct< T, Eigen::Matrix< S,-1,-1 > > | Struct to hold static function for promoting underlying scalar types |
| stan::math::promote\_scalar\_struct< T, std::vector< S > > | Struct to hold static function for promoting underlying scalar types |
| stan::math::promote\_scalar\_struct< T, T > | Struct to hold static function for promoting underlying scalar types |
| stan::math::promote\_scalar\_type< T, S > | Template metaprogram to calculate a type for converting a convertible type |
| stan::math::promote\_scalar\_type< T, Eigen::Matrix< S, 1, Eigen::Dynamic > > | Template metaprogram to calculate a type for a row vector whose underlying scalar is converted from the second template parameter type to the first |
| stan::math::promote\_scalar\_type< T, Eigen::Matrix< S, Eigen::Dynamic, 1 > > | Template metaprogram to calculate a type for a vector whose underlying scalar is converted from the second template parameter type to the first |
| stan::math::promote\_scalar\_type< T, Eigen::Matrix< S, Eigen::Dynamic, Eigen::Dynamic > > | Template metaprogram to calculate a type for a matrix whose underlying scalar is converted from the second template parameter type to the first |
| stan::math::promote\_scalar\_type< T, std::vector< S > > | Template metaprogram to calculate a type for a container whose underlying scalar is converted from the second template parameter type to the first |
| stan::math::promoter< F, T > |  |
| stan::math::promoter< Eigen::Matrix< F, R, C >, Eigen::Matrix< T, R, C > > |  |
| stan::math::promoter< Eigen::Matrix< T, R, C >, Eigen::Matrix< T, R, C > > |  |
| stan::math::promoter< std::vector< F >, std::vector< T > > |  |
| stan::math::promoter< std::vector< T >, std::vector< T > > |  |
| stan::math::promoter< T, T > |  |
| stan::mcmc::ps\_point |  |
| stan::mcmc::dense\_e\_point |  |
| stan::mcmc::diag\_e\_point |  |
| stan::mcmc::unit\_e\_point |  |
| stan::gm::range |  |
| stan::io::reader< T > | A stream-based reader for integer, scalar, vector, matrix and array data types, with Jacobian calculations |
| stan::gm::reject\_statement |  |
| stan::gm::remove\_loop\_identifier |  |
| stan::gm::unscope\_variables::result< T1, T2 > |  |
| stan::gm::binary\_op\_expr::result< T1, T2, T3, T4, T5 > |  |
| stan::gm::add\_conditional\_body::result< T1, T2 > |  |
| set\_double\_range\_upper::result< T1, T2, T3, T4 > |  |
| validate\_int\_data\_expr::result< T1, T2, T3, T4, T5 > |  |
| stan::gm::add\_expression\_dimss::result< T1, T2, T3, T4 > |  |
| stan::gm::logical\_negate\_expr::result< T1, T2, T3 > |  |
| copy\_square\_cholesky\_dimension\_if\_necessary::result< T1 > |  |
| stan::gm::validate\_expr\_type3::result< T1, T2 > |  |
| stan::gm::set\_fun\_type3::result< T1, T2 > |  |
| stan::gm::addition\_expr3::result< T1, T2, T3 > |  |
| stan::gm::subtraction\_expr3::result< T1, T2, T3 > |  |
| stan::gm::set\_fun\_type2::result< T1, T2 > |  |
| stan::gm::validate\_non\_void\_arg\_function::result< T1, T2, T3 > |  |
| stan::gm::set\_void\_function::result< T1, T2, T3, T4 > |  |
| stan::gm::set\_allows\_sampling\_origin::result< T1, T2, T3 > |  |
| stan::gm::validate\_declarations::result< T1, T2, T3, T4 > |  |
| stan::gm::add\_function\_signature::result< T1, T2, T3, T4, T5 > |  |
| stan::gm::validate\_return\_type::result< T1, T2, T3 > |  |
| stan::gm::elt\_division\_expr::result< T1, T2, T3 > |  |
| stan::gm::scope\_lp::result< T1 > |  |
| stan::gm::add\_conditional\_condition::result< T1, T2, T3, T4 > |  |
| set\_int\_range\_lower::result< T1, T2, T3, T4 > |  |
| stan::gm::division\_expr::result< T1, T2, T3 > |  |
| stan::gm::validate\_return\_allowed::result< T1, T2, T3 > |  |
| stan::gm::validate\_void\_return\_allowed::result< T1, T2, T3 > |  |
| stan::gm::validate\_assignment::result< T1, T2, T3, T4 > |  |
| stan::gm::set\_fun\_type::result< T1, T2 > |  |
| stan::gm::validate\_sample::result< T1, T2, T3 > |  |
| stan::gm::expression\_as\_statement::result< T1, T2, T3 > |  |
| stan::gm::unscope\_locals::result< T1, T2 > |  |
| stan::gm::add\_while\_condition::result< T1, T2, T3 > |  |
| stan::gm::add\_while\_body::result< T1, T2 > |  |
| stan::gm::add\_loop\_identifier::result< T1, T2, T3, T4 > |  |
| stan::gm::remove\_loop\_identifier::result< T1, T2 > |  |
| stan::gm::validate\_int\_expr2::result< T1, T2, T3 > |  |
| stan::gm::validate\_allow\_sample::result< T1, T2, T3 > |  |
| stan::gm::validate\_non\_void\_expression::result< T1, T2, T3 > |  |
| stan::gm::validate\_integrate\_ode::result< T1, T2, T3, T4 > |  |
| stan::gm::set\_fun\_type\_named::result< T1, T2, T3, T4, T5 > |  |
| stan::gm::exponentiation\_expr::result< T1, T2, T3, T4, T5 > |  |
| stan::gm::multiplication\_expr::result< T1, T2, T3 > |  |
| stan::gm::modulus\_expr::result< T1, T2, T3, T4 > |  |
| stan::gm::left\_division\_expr::result< T1, T2, T3 > |  |
| stan::gm::elt\_multiplication\_expr::result< T1, T2, T3 > |  |
| stan::gm::negate\_expr::result< T1, T2, T3, T4 > |  |
| stan::gm::transpose\_expr::result< T1, T2 > |  |
| stan::gm::set\_var\_type::result< T1, T2, T3, T4 > |  |
| stan::gm::validate\_int\_expr3::result< T1, T2 > |  |
| add\_var::result< T1, T2, T3, T4, T5, T6 > |  |
| validate\_identifier::result< T1, T2 > |  |
| validate\_decl\_constraints::result< T1, T2, T3, T4 > |  |
| empty\_range::result< T1 > |  |
| validate\_int\_expr::result< T1, T2, T3 > |  |
| validate\_double\_expr::result< T1, T2 > |  |
| set\_double\_range\_lower::result< T1, T2, T3, T4 > |  |
| stan::gm::add\_fun\_var::result< T1, T2, T3, T4 > |  |
| set\_int\_range\_upper::result< T1, T2, T3, T4 > |  |
| stan::gm::return\_statement |  |
| stan::return\_type< T1, T2, T3, T4, T5, T6 > | Metaprogram to calculate the base scalar return type resulting from promoting all the scalar types of the template parameters |
| stan::gm::sample |  |
| stan::mcmc::sample |  |
| Eigen::internal::scalar\_product\_traits< double, stan::agrad::var > | Scalar product traits override for Eigen for automatic gradient variables |
| Eigen::internal::scalar\_product\_traits< stan::agrad::var, double > | Scalar product traits override for Eigen for automatic gradient variables |
| stan::scalar\_type< T > | Metaprogram structure to determine the base scalar type of a template argument |
| stan::scalar\_type< Eigen::Matrix< T, Eigen::Dynamic, Eigen::Dynamic > > |  |
| stan::scalar\_type< T \* > |  |
| stan::scalar\_type\_pre< T > | Metaprogram structure to determine the type of first container of the base scalar type of a template argument |
| stan::gm::scope\_lp |  |
| stan::math::seq\_view< T, S > |  |
| stan::math::seq\_view< double, std::vector< int > > |  |
| stan::math::seq\_view< T, Eigen::Matrix< S, 1, Eigen::Dynamic > > |  |
| stan::math::seq\_view< T, Eigen::Matrix< S, Eigen::Dynamic, 1 > > |  |
| stan::math::seq\_view< T, Eigen::Matrix< S, Eigen::Dynamic, Eigen::Dynamic > > |  |
| stan::math::seq\_view< T, std::vector< S > > |  |
| stan::math::seq\_view< T, std::vector< std::vector< T > > > |  |
| stan::math::seq\_view< T, std::vector< T > > |  |
| stan::gm::set\_allows\_sampling\_origin |  |
| set\_double\_range\_lower |  |
| set\_double\_range\_upper |  |
| stan::gm::set\_fun\_type |  |
| stan::gm::set\_fun\_type2 |  |
| stan::gm::set\_fun\_type3 |  |
| stan::gm::set\_fun\_type\_named |  |
| set\_int\_range\_lower |  |
| set\_int\_range\_upper |  |
| stan::gm::set\_var\_type |  |
| stan::gm::set\_void\_function |  |
| Eigen::internal::significant\_decimals\_default\_impl< stan::agrad::var, false > | Implemented this for printing to stream |
| stan::size\_of\_helper< T, is\_vec > |  |
| stan::size\_of\_helper< T, true > |  |
| stan::memory::stack\_alloc | An instance of this class provides a memory pool through which blocks of raw memory may be allocated and then collected simultaneously |
| stan::io::stan\_csv |  |
| stan::io::stan\_csv\_adaptation |  |
| stan::io::stan\_csv\_metadata |  |
| stan::io::stan\_csv\_reader | Reads from a Stan output csv file |
| stan::io::stan\_csv\_timing |  |
| stan::gm::statement |  |
| stan::gm::statements |  |
| **static\_visitor** |  |
| data\_only\_expression |  |
| stan::gm::contains\_nonparam\_var |  |
| stan::gm::contains\_var |  |
| stan::gm::expression\_type\_vis |  |
| stan::gm::is\_nil\_op |  |
| stan::gm::is\_no\_op\_statement\_vis |  |
| stan::gm::name\_vis |  |
| stan::gm::returns\_type\_vis |  |
| stan::math::store\_type< T > |  |
| stan::math::store\_type< double > |  |
| stan::math::store\_type< Eigen::Matrix< S, 1, Eigen::Dynamic > > |  |
| stan::math::store\_type< Eigen::Matrix< S, Eigen::Dynamic, 1 > > |  |
| stan::math::store\_type< Eigen::Matrix< S, Eigen::Dynamic, Eigen::Dynamic > > |  |
| stan::math::store\_type< int > |  |
| stan::math::store\_type< S > |  |
| stan::math::store\_type< std::vector< int > > |  |
| stan::math::store\_type< std::vector< S > > |  |
| stan::math::store\_type< std::vector< std::vector< T > > > |  |
| stan::math::store\_type< std::vector< T > > |  |
| stan::gm::subtraction\_expr3 |  |
| stan::common::recorder::sum\_values |  |
| stan::gm::transpose\_expr |  |
| stan::gm::type\_name< T > |  |
| stan::gm::type\_name< bool > |  |
| stan::gm::type\_name< double > |  |
| stan::gm::type\_name< int > |  |
| stan::gm::type\_name< std::string > |  |
| stan::gm::type\_name< unsigned int > |  |
| stan::gm::unary\_op |  |
| stan::gm::unscope\_locals |  |
| stan::gm::unscope\_variables |  |
| stan::gm::validate\_allow\_sample |  |
| stan::gm::validate\_assignment |  |
| validate\_decl\_constraints |  |
| stan::gm::validate\_declarations |  |
| validate\_double\_expr |  |
| stan::gm::validate\_expr\_type3 |  |
| validate\_identifier |  |
| validate\_int\_data\_expr |  |
| validate\_int\_expr |  |
| stan::gm::validate\_int\_expr2 |  |
| stan::gm::validate\_int\_expr3 |  |
| stan::gm::validate\_integrate\_ode |  |
| stan::gm::validate\_non\_void\_arg\_function |  |
| stan::gm::validate\_non\_void\_expression |  |
| stan::gm::validate\_return\_allowed |  |
| stan::gm::validate\_return\_type |  |
| stan::gm::validate\_sample |  |
| stan::gm::validate\_void\_return\_allowed |  |
| stan::math::value\_type< T > | Primary template class for metaprogram to compute the type of values stored in a container |
| stan::math::value\_type< const T > | Template class for metaprogram to compute the type of values stored in a constant container |
| stan::math::value\_type< Eigen::Matrix< T, R, C > > | Template metaprogram defining the type of values stored in an Eigen matrix, vector, or row vector |
| stan::math::value\_type< std::vector< T > > | Template metaprogram class to compute the type of values stored in a standard vector |
| stan::common::recorder::values< InternalVector > |  |
| stan::agrad::var | Independent (input) and dependent (output) variables for gradients |
| stan::io::var\_context | A `var_reader` reads array variables of integer and floating point type by name and dimension |
| stan::io::dump | Represents named arrays with dimensions |
| stan::json::json\_data | A `json_data` is a `var_context` object that represents a set of named values which are typed to either `double` or `int` and can be either scalar value or a non-empty array of values of any dimensionality |
| stan::common::var\_context\_factory< VARCON > |  |
| stan::common::var\_context\_factory< stan::io::dump > |  |
| stan::common::dump\_factory |  |
| stan::gm::var\_decl |  |
| stan::gm::variable |  |
| stan::gm::variable\_dims |  |
| stan::gm::variable\_map |  |
| stan::VectorView< T, is\_array, throw\_if\_accessed > | VectorView is a template metaprogram that takes its argument and allows it to be used like a vector |
| stan::VectorView< const double, false, false > |  |
| stan::VectorView< const T, is\_array, throw\_if\_accessed > | VectorView that has const correctness |
| stan::VectorView< double \*, stan::is\_vector< T1 >::value, stan::is\_constant\_struct< T1 >::value > |  |
| stan::VectorView< double \*, stan::is\_vector< T2 >::value, stan::is\_constant\_struct< T2 >::value > |  |
| stan::VectorView< double \*, stan::is\_vector< T3 >::value, stan::is\_constant\_struct< T3 >::value > |  |
| stan::VectorView< double \*, stan::is\_vector< T4 >::value, stan::is\_constant\_struct< T4 >::value > |  |
| stan::VectorView< double \*, stan::is\_vector< T5 >::value, stan::is\_constant\_struct< T5 >::value > |  |
| stan::VectorView< double \*, stan::is\_vector< T6 >::value, stan::is\_constant\_struct< T6 >::value > |  |
| stan::VectorViewMvt< T, is\_array, throw\_if\_accessed > |  |
| stan::VectorViewMvt< const T, is\_array, throw\_if\_accessed > | VectorViewMvt that has const correctness |
| stan::gm::visgen | Generic visitor with output for extension |
| stan::gm::constrained\_param\_names\_visgen |  |
| stan::gm::dump\_member\_var\_visgen |  |
| stan::gm::expression\_visgen |  |
| stan::gm::generate\_init\_vars\_visgen |  |
| stan::gm::generate\_init\_visgen |  |
| stan::gm::generate\_local\_var\_init\_nan\_visgen |  |
| stan::gm::init\_local\_var\_visgen |  |
| stan::gm::local\_var\_decl\_visgen |  |
| stan::gm::member\_var\_decl\_visgen |  |
| stan::gm::printable\_visgen |  |
| stan::gm::set\_param\_ranges\_visgen |  |
| stan::gm::statement\_visgen |  |
| stan::gm::unconstrained\_param\_names\_visgen |  |
| stan::gm::validate\_transformed\_params\_visgen |  |
| stan::gm::validate\_var\_decl\_visgen |  |
| stan::gm::var\_resizing\_visgen |  |
| stan::gm::var\_size\_validating\_visgen |  |
| stan::gm::write\_array\_vars\_visgen |  |
| stan::gm::write\_array\_visgen |  |
| stan::gm::write\_csv\_header\_visgen |  |
| stan::gm::write\_csv\_vars\_visgen |  |
| stan::gm::write\_csv\_visgen |  |
| stan::gm::write\_dims\_visgen |  |
| stan::gm::write\_param\_names\_visgen |  |
| stan::prob::welford\_covar\_estimator |  |
| stan::prob::welford\_var\_estimator |  |
| stan::gm::while\_statement |  |
| stan::io::writer< T > | A stream-based writer for integer, scalar, vector, matrix and array data types, which transforms from constrained to a sequence of constrained variables |


---

[ Stan Home Page ]

*© 2011–2014,
Stan Development Team.*
